# Skin infection by *Mycobacterium marinum* – diagnostic and therapeutic challenge^⋆^

**DOI:** 10.1016/j.abd.2021.03.013

**Published:** 2022-04-12

**Authors:** Angélica Seidel, Daniel Holthausen Nunes, Camilo Fernandes, Gabriella Di Giunta Funchal

**Affiliations:** a Hospital Universitário Professor Polydoro Ernani de São Thiago, Universidade Federal de Santa Catarina, Florianópolis, SC, Brazil; b Hospital Nereu Ramos – Secretaria de Estado de Saúde Santa Catarina, Florianópolis, SC, Brazil

**Keywords:** Bacteria, Infections, Skin

## Abstract

The number of skin infections caused by atypical mycobacteria has increased in recent decades. They usually appear after contact with wounds and interruptions in the integrity of the skin. The present report describes a case of cutaneous infection by *Mycobacterium marinum*, in a young, immunocompetent patient, with a prolonged evolution, diagnosed through a skin lesion culture (from a spindle biopsy of the skin). The patient was treated with multidrug therapy, including clarithromycin, doxycycline, and rifampicin, due to the lesion extent, with satisfactory results. A brief review of the literature is also provided.

## Introduction

The number of skin infections caused by non-tuberculous mycobacteria (NTM) has been increasing in recent decades.^1^ There are an average of 130 species and of these, 1/3 can cause infection in humans.^1^ Some have a worldwide distribution, such as the fast-growing ones, while others predominate in Africa and Australia, causing endemic skin infection.^1^

The cutaneous disease usually arises after the contact of interruptions in the integrity of the skin (such as wounds caused by trauma or surgery)^1^, ^2^ with water or other contaminated products, or by hematogenous spread, especially in immunocompromised patients.^1^

Its diagnosis is difficult and requires clinical suspicion due to a history of local trauma with subsequent contamination, which is confirmed by culture of the lesion.^1^, ^3^

The treatment is prolonged. When possible, it should be based on the susceptibility rates of the bacteria causing the infection.^1^, ^3^ However, in the absence of an antibiogram and in cases of diagnosis obtained through molecular tests, it is recommended to guide the choice of treatment according to the group of mycobacteria responsible for the disease.^3^

The authors of the present study report below a case of cutaneous infection by *Mycobacterium marinum* addressing its diagnostic difficulty and therapeutic management.

## Case report

**Figure 1:**
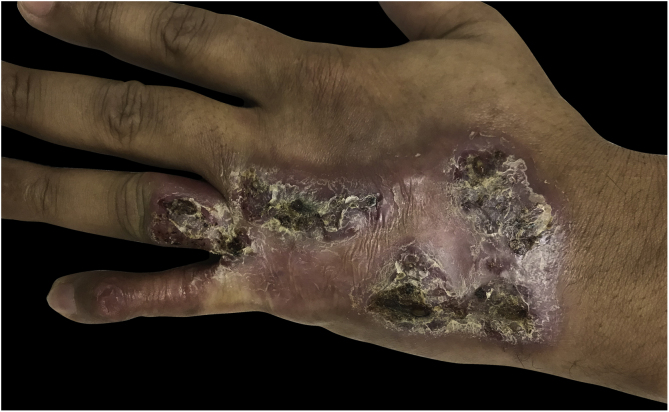
Erythematous and verrucous plaque, with nodules and crusts, located on the 4th and 5th fingers, in addition to the dorsal aspect of the left hand.

**Figure 2:**
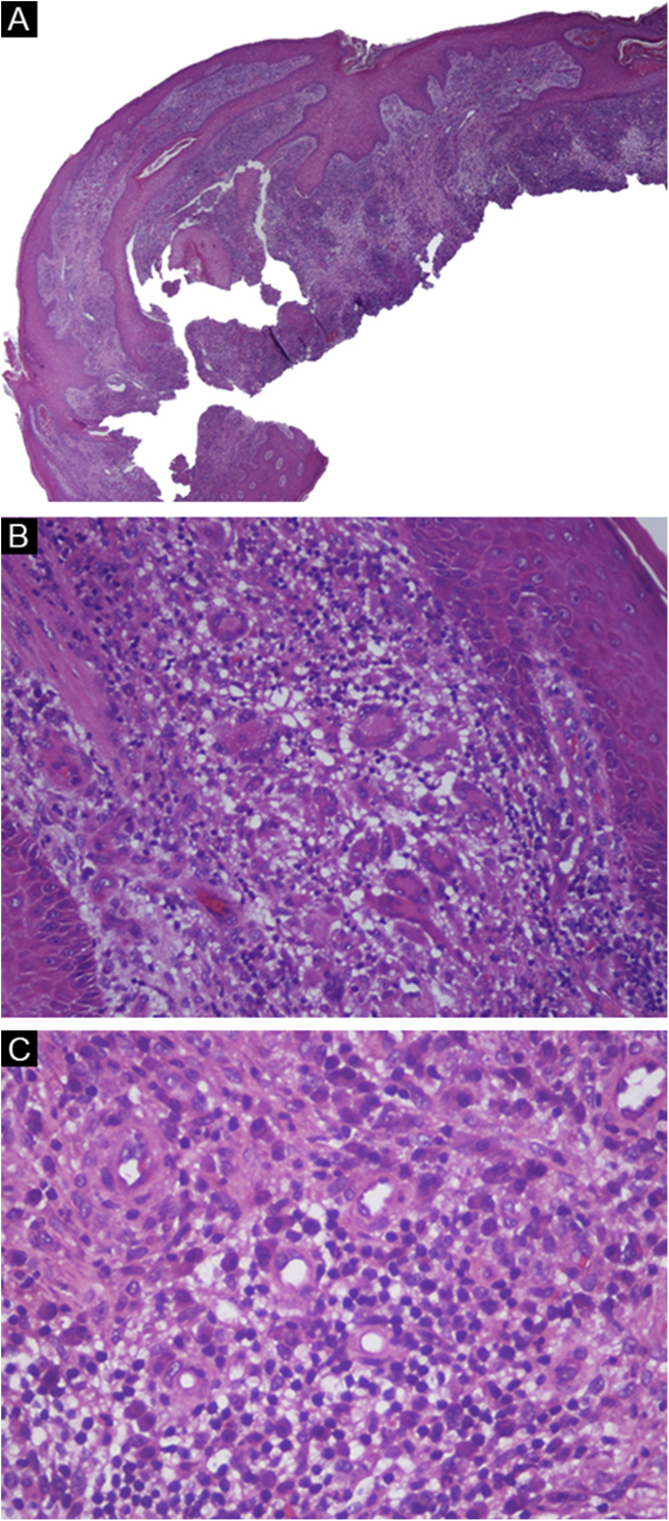
Histopathological analysis of skin biopsy. (A) Pseudoepitheliomatous hyperplasia, (Hematoxylin & eosin, ×20). (B) Multinucleated giant cells, (Hematoxylin & eosin, ×200). (C) Plasma cells, (Hematoxylin & eosin, ×400).

**Figure 3:**
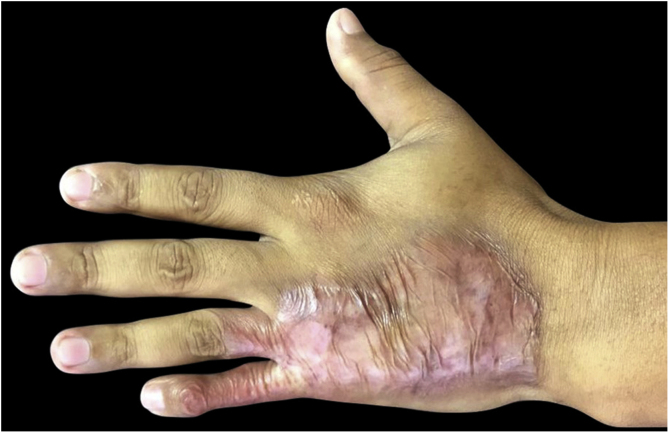
Clinical improvement after 4 months of treatment.

A 36-year-old healthy male patient, who lived in the city of Florianópolis, state of Santa Catarina, Brazil, and worked as a construction worker, had the occasional habit of fishing in mangroves. He mentioned the appearance of a lesion on the 5^th^ left finger (LF) 15 years before, after trauma with a perforating-cutting tool. It developed a papule and exudation, with subsequent progression of the lesion to the dorsal aspect of the hand, forming an erythematous-verrucous plaque with nodules and crusts and evident atrophy of the 5^th^ finger (Fig. 1). He denied systemic symptoms. The patient was submitted to punch biopsies, whose histopathological analysis suggested an infectious process but with negative cultures. The patient received several empirical treatments without improvement. A tomography of the left hand ruled out bone involvement and the serologies were negative. The patient was submitted to another spindle biopsy of the lesion and histopathology disclosed a difuse lymphomononuclear infiltrate rich in plasma cells, neutrophils (Fig. 2), while culture (in solid Lowenstein-Jensen medium) showed *Mycobacterium marinum* growth. The treatment with clarithromycin, doxycycline, and rifampicin was started, due to the lesion extension, with a good response (Fig. 3).

## Discussion

*Mycobacterium marinum* lives in aquatic environments, preferably untreated saltwater.^1^, ^3^ Since 1954, its infection has been known as “swimming pool” or “fish tank” granuloma.^1^, ^3^ It is an uncommon infection, with an incidence of 0.04 to 0.27/100,000 inhabitants.^3^

It predominates in the second and third decades of life, with no sex predilection.^3^ Regarding the incidence in immunosuppressed and immunocompetent patients,^3^, ^4^ studies are still scarce to confirm a predominance in either group since the data in the literature remain discordant. Its incubation period lasts two to eight weeks.^1^

The lesion presents as a single, purplish papule, plaque, or nodule, usually on the extremities (such as the dorsal region of the hands or feet).^1^, ^2^, ^3^, ^4^ It may progress to suppuration, ulceration, or even a debilitating, disseminated infection.^1^, ^3^

Clinical suspicion is necessary for the diagnosis to be made. A biopsy with histopathological analysis and culture of the lesion should be performed.^3^, ^4^ The anatomopathological analysis reveals a granulomatous and suppurative process in the dermis, and the culture identifies the bacillus in a few cases.^3^

The treatment depends on the species, extent of the lesion, and the patient's immunological status.^3^, ^4^ Mild cases with superficial infection can be treated with monotherapy (clarithromycin, minocycline, doxycycline, trimethoprim/sulfamethoxazole). Whereas patients with severe and long-term conditions require the association of two or more drugs (rifampicin, ethambutol, macrolides, trimethoprim/sulfamethoxazole) and eventually resection and debridement.^1^, ^2^, ^3^

This case report describes a case of skin infection caused by *Mycobacterium marinum* with a long evolution and difficult diagnosis, emphasizing the importance of clinical suspicion, and of performing multiple biopsies of the lesion for culture and histopathological confirmation.

## Financial support

None declared.

## Authors' contributions

Angélica Seidel: Design and planning of the study; drafting and editing of the manuscript; collection, analysis, and interpretation of data; critical review of the literature.

Daniel Holthausen Nunes: Approval of the final version of the manuscript; critical review of the manuscript; intellectual participation in the propaedeutic and/or therapeutic conduct of the studied cases.

Camilo Fernandes: Approval of the final version of the manuscript; critical review of the manuscript; intellectual participation in the propaedeutic and/or therapeutic conduct of the studied cases.

Gabriella Di Giunta Funchal: Approval of the final version of the manuscript; critical review of the manuscript; design and planning of the study.

## Conflicts of interest

None declared.
